# Novel p53 reactivators that are synergistic with olaparib for the treatment of gynecologic cancers with mutant p53

**DOI:** 10.1016/j.tranon.2025.102522

**Published:** 2025-09-06

**Authors:** Lane E. Smith, Jamie L. Padilla, Angelina Licor, Mara P. Steinkamp, Irina V. Lagutina, Yan Guo, Eric J. Devor, Vernon S. Pankratz, Annahita Sallmyr, Olufunmilola M. Oyebamiji, Jun-yong Choe, Geneva L. Williams, Kimberly K. Leslie

**Affiliations:** a The University of New Mexico, Albuquerque, NM, USA; b St John’s University, Queens, NY, USA; c The University of Miami, Miami, FL, USA; d The University of Iowa, Iowa City, IA, USA

**Keywords:** Ovarian cancer, Endometrial cancer, HO-3867, APR-246, Prima-1met, Olaparib, P53, BRCA

## Abstract

Ovarian and endometrial cancers frequently harbor a mutation in the tumor suppressor gene *TP53*, which occurs in over 90 % of ovarian cancers and in the most aggressive endometrial cancers. The normal tumor suppressive functions of p53 are disrupted, resulting in unregulated cell growth and therapeutic resistance to standard treatments including chemotherapy and PARP inhibitors. Hence, a novel therapeutic strategy is urgently needed for p53 mutant gynecologic cancers, and we propose that converting mutant p53 to a wild type conformation and restoring its tumor suppressive functions has the potential to greatly improve treatment. In this study, we investigated the effects of two purported p53 reactivators, HO-3867 and APR-246, on cell proliferation via half-maximal inhibitory concentration (IC50) analyses using CyQUANT DNA measurements, tumor growth *in vivo* and gene expression by bulk RNA sequencing in gynecologic cancer cell lines that harbor oncogenic mutations in p53. We also tested these compounds in combination with the PARP inhibitor olaparib. We found that HO-3867 was very effective in inhibiting growth, with IC50 values in the low micromolar range. Importantly, HO-3867 was synergistic with olaparib treatment in five cell lines studied *in vitro* as well as *in vivo* in a xenograft model of high grade serous ovarian cancer. RNA sequencing data suggest that HO-3867 is acting through both p53-independent and p53-dependent pathways resulting in inhibition of DNA repair pathways including homologous recombination in p53 mutant cancer cells.

**Significance:** The development of resistance to PARP inhibitors is a major problem and a cause of treatment failures in advanced gynecologic cancers, and we show that adding a p53 reactivator such as HO-3867 enhances the efficacy of PARP inhibitors in p53-mutant cancer models.

## Highlights

• Therapeutic agents that target mutant p53 and reactivate pro-apoptotic functionality have potential to improve outcomes in cancer patients.
• These studies in relevant preclinical cell and animal models demonstrate that HO-3867 and APR-246, two p53 reactivator agents, are active and can synergize with PARP inhibitors as a novel therapeutic approach for future testing.
• HO-3867 is shown to induce apoptosis, ER stress, and DNA damage in several gynecologic cancer cell lines.

## Introduction

Endometrial cancer is the most common gynecologic malignancy in women, accounting for >66,000 cases and 13,000 deaths in the United States each year. Unfortunately, both incidence and mortality rates are rising for endometrial cancer [1], and in a large retrospective analysis of cancer patient outcomes, endometrial cancer was the only cancer type in which patients had a worse 5-year survival rate in this decade compared to 1975 [2]. While deaths from endometrial cancer surpassed those from ovarian cancer just in the past year, ovarian cancer remains the most lethal cancer in women with an average 5-year survival rate of only 50 % [3]. The majority of high grade serous endometrial and ovarian cancers harbor mutated *TP53*. These are aggressive, often lethal tumors that are an important cause of the significantly rising morbidity and mortality from endometrial cancer and are associated with ovarian cancer recurrence. Thus, a strategy is urgently needed to combat tumorigenesis driven by altered p53.

The severity of p53 dysfunction is determined by the type of mutations in the gene *TP53*, which are classified as loss of function (LOF), frameshift, insertion/deletion, silent, or missense. Missense mutations include the gain-of-function (GOF) oncogenic forms, known as “hotspot” mutations, which commonly occur across all cancer types [4]. GOF mutations occur within the DNA binding domain (DBD) of p53 and cause loss of transcriptional activity either by directly disrupting DNA binding or by destabilizing the protein and preventing zinc binding which is required for p53 activation [5]. Further, GOF mutants can suppress and/or activate additional target genes/proteins, giving the tumor a growth advantage beyond the p53 null state [[6], [7], [8]].

Many tumors acquire altered p53 as an early step in tumorigenesis. Loss of wild type (WT) p53 function affects a myriad of critical cell functions and thus, reinstating WT p53 function is an intriguing method to target these cancers. Though p53 is one of the most extensively studied proteins, relatively few agents have been shown to bind mutant p53 and reconfigure its structure to a WT state. Such agents, sometimes referred to as “p53 reactivators,” are proposed to bind to misfolded p53 and re-establish the normal DNA binding and transcriptional activity of the protein [9], leading to apoptosis in cells with extensive genomic instability [[10], [11], [12]]. APR-246, also known as PRIMA-1^Met^, is one of two p53 reactivators that has entered clinical trials [13], though its complete targets and methods of action are not completely understood. Previous studies have shown the drug is converted to a methylene quinuclidinone (MQ) compound which covalently binds to the p53 core domain [14,15], and synergistic effects with chemotherapy drugs have been observed in preclinical models of ovarian cancer [16]. APR-246 received breakthrough therapy, orphan drug and fast track designations from the FDA for myelodysplastic syndrome (MDS) and orphan drug designation from the European Commission for MDS, acute myeloid leukemia (AML) and ovarian cancer [17,18]. Significant clinical activity was recently documented in combination with azacytidine in patients with *TP53* mutant MDS [19,20]; however, APR-246 has not yet gained FDA approval [21]. Another compound, HO-3867, an analogue of curcumin, has been shown to covalently bind mutant forms of p53 near the DNA binding domain and has been evaluated in preclinical studies [9,22], though it is not known to which extent the compound is able to bind different types of p53 mutants. In addition to binding p53, HO-3867 has been shown to bind to Signal Transducer and Activator of Transcription 3 (STAT3) through direct covalent binding in ovarian cancer [9,22]. Further, HO-3867 was shown to restore suppression of the placenta-specific protein 1 (PLAC1), a direct target of WT p53 [23]. Overall, p53 reactivators are a promising class of targeted agents, but a more thorough understanding of their mechanisms of action is needed.

While the development of poly (ADP-ribose) polymerase (PARP) inhibitors for maintenance therapy or adjuvant settings to combat cancers has improved progression-free survival (PFS), even these targeted agents place patients at risk for future MDS and often fail due to cancer cells acquiring resistance [24]. The co-mutation of *TP53* and genes associated with homologous recombination repair (HRR) such as *BRCA1/2* is a common and high-risk tumor genotype in gynecologic malignancies [25]. There is selective pressure for cancer cells with an inactivating *BRCA* mutation to also mutate *TP53*, as loss of the high-fidelity HRR pathway results in an accumulation of genomic instability and induction of apoptosis by p53. Expressing WT p53 in BRCA mutated or HRR deficient cells enhances cell death, and creating BRCA deficient models requires the co-mutation of *TP53* [26,27]. Thus, p53 reactivator agents such as HO-3867 and APR-246 have the potential for improved treatment specifically for cancers with missense *TP53* mutations and HRR deficiency. We hypothesize that BRCA protein loss is synthetically lethal with WT p53 function and thus expect a synergistic effect between PARP inhibitors and p53 reactivators in p53/BRCA mutated tumors.

In these studies, we explored the hypothesis that p53 reactivator agents will be effective in preclinical studies of ovarian and endometrial cancer cells and assessed the synergy between these agents and the PARP inhibitor olaparib. We report the significant growth limiting effects of these strategies and provide mechanistic data underscoring how these agents work in high-risk gynecologic cancer cells with various *TP53* and *BRCA* mutations. The expression of tumor suppressive target transcripts of WT p53 were reinstated after treatment with APR-246 and HO-3867. Further, synergy between HO-3867 and olaparib was documented in many of the gynecologic cancer cells lines studied *in vitro* as well as *in vivo* in a xenograft model. We also identified mechanisms of HO-3867 action beyond directly targeting p53, including other pro-apoptotic pathways, the inhibition of DNA repair and induction of the DNA damage response, and immunomodulatory pathways, thereby broadening the potential use of this compound even in p53 null cancers.

## Methods

### Cell lines and chemicals

PEO1 cells were obtained from Dr. Elizabeth Swisher at the University of Washington. PEO4 cells were purchased from Sigma Aldrich (Cat. #10,032,309) as were COV362 (Cat. #07,071,910). Ishikawa and Hec50co cells were acquired from Dr. Erlio Gurpide, New York University. OVCAR3 cells were acquired from ATCC (Cat. #HTB-161). All cell lines were validated by STR genotyping (Bio-Synthesis, Inc., Lewisville, TX USA) and tested for *Mycoplasma* contamination (MycoStrip; Invivogen, #rep-mys-100) prior to experiments. Ishikawa, Hec50co, COV362, PEO1, and PEO4 cells were cultured in DMEM (Gibco; Thermo Fisher Scientific) supplemented with 10 % fetal bovine serum (FBS) and 1 % penicillin-streptomycin (Gibco; Thermo Fisher Scientific). Glutamine (2 mM; Gibco, Cat. #25,030,081) was added to COV362 cell media. OVCAR3 cells were cultured in RPMI 1640 (Gibco) supplemented with 20 % FBS, 1 % MEM NEAA (Quality Biological; Cat. #116–078–721) and 1 % pen-strep. Cells were passaged fewer than ten times over the duration of experiments and kept at 37 °C in 5 % CO_2_. For cell studies, olaparib (#10,621, CAS No 763,113–22–0), HO-3867 (Cat. #21,581, CAS No 1,172,133–28–6) and APR-246 (Cat. #9,000,487, CAS No 5291–32–7) were purchased from Cayman Chemical (Ann Arbor, MI). For xenograft models, HO-3867 was purchased in bulk from Selleck (Cat. #S7501) and sent to Inotiv (Madison, WI) where it was incorporated into the Teklad 2020 base rodent diet (Cat. #2920X) at a concentration of 100 mg/kg.

### Western blotting

Cells were cultured in their appropriate media as described above. For treated cells, a concentration of 4 μm HO-3867 or 10 μM olaparib was added to adherent cells for 24 h. Trypsin was added to plates to collect cells and whole-cell lysates were prepared using RIPA buffer (Thermo Scientific; Cat.#89,900) with Halt protease inhibitor added (Thermo Scientific; Cat.#78,430). Protein concentrations were determined using the Qubit Protein Broad Range Assay Kit (Invitrogen; Cat.#A50668). Lysates were denatured in NuPage Loading Buffer (Invitrogen; Cat.#NP0007) and 50µ g was loaded onto a 10 % NuPage gel (Invitrogen; Cat.#NP0322). The separated proteins were transferred to a PVDF membrane (Thermo Scientific; Cat. #88,585). The membrane was blocked with 5 % dry milk in 1X TBST for 1h at room temperature and incubated overnight at 4 °C with the following primary antibodies: p53 (Cell Signaling Technology, Cat.#2527; 1:1000), STAT3 (Cell Signaling Technology, Cat.#4904S; 1:1000), or 53BP1 (Cell Signaling Technology, Cat.#4937S; 1:1000). The membranes were washed, and HRP-linked secondary antibodies diluted in TBST were added to the membranes: anti-rabbit IgG (Cell Signaling Technology, #7074, 1:3000), H3 (Cell Signaling Technology, #12,648, 1:1000), or GAPDH loading controls (Santa Cruz Biotechnology; sc-47,724; 1:1000) were included in each experiment. Membranes were incubated for 1h at room temperature and washed. The bound antibodies were detected using Pierce™ ECL Western Blotting Substrate (Thermo Scientific; #32,109), visualized on the Li-COR System, and analyzed using Image Studio Version 5.2.

### Cell proliferation and apoptosis assays

For cell proliferation studies, the optimal seeding density for each cell line was first obtained by assessing cell confluence after 98 h at various seeding densities (1250, 2500, 5000, and 10,000 cells/well) on a 96-well plate. Cells were seeded at their optimal density in 100 µL of complete growth media and grown for 24 h. Drugs or DMSO controls were then added to 100 µL of fresh media and added to triplicate wells. Plates were incubated for 72 h, and the total DNA content in each well was measured using the CyQUANT NF Cell Proliferation Assay (Thermo Fisher, #C35006) according to the manufacturer’s protocol. Fluorescence values were determined on the Wallac 1420 multilabel counter (PerkinElmer). For combination studies, one drug was held constant at its estimated IC50 value while the second drug was added in increasing concentrations to the same wells. For internal controls, the second drug was added alone in identical concentrations as used in combination wells. The first drug was also added alone in triplicate wells to confirm the IC50 concentration. All treatments were performed in triplicate, and all experiments were independently repeated to confirm results. To assess caspase-mediated apoptosis, COV362 cells were seeded into 96 well plates and allowed to grow for 24 h. HO-3867 or DMSO control was then added to appropriate wells. CellEvent™ Caspase-3/7 Detection Reagent (Thermo Fisher, #C10423) was added at a 5 µM final concentration to each well and the plates were imaged on the Incucyte Live-Cell Analysis System (Sartorius). Four replicate wells were used for each treatment and experiments were independently repeated to confirm results.

### Cell and xenograft mouse studies

OVCAR3 cells were obtained under an MTA from the National Cancer Institute’s (NCI’s) Biological Testing Branch of the Cancer Therapeutics Program, DCTD Tumor (and Cell) Repository. OVCAR3 cells were transduced with Red F-luc-GFP (CLS960003, Revvity) and sorted for GFP. OVCAR3-RLuc ovarian cancer cells were cultured in RPMI 1640 (Corning) supplemented with 10 % fetal bovine serum (FBS), 2 mM l-glutamine, 100 U/ml penicillin and 100 μg/ml streptomycin. All animal experiments were performed under an approved protocol reviewed by the Institutional Animal Care and Use Committee (IACUC) of the University of New Mexico Health Science Center. 5 × 10^6^ OVCAR3-RLuc cells were intraperitoneally injected into 7–week old NSG female mice (RRID:IMSR_JAX:005,557). Intraperitoneal tumor burden was measured two weeks after OVCAR3-RLuc injection and quantified by bioluminescent imaging on the IVIS Spectrum *in vivo* imaging system (Perkin Elmer). Mice with no or low tumor burden were excluded from the study. Mice with a minimum tumor burden of 1.4 × 10^9^ photons/*sec* total flux were evenly distributed among four treatment groups. Mice were treated daily for five weeks with olaparib (IP, 50 mg/kg), HO-3867 (chow, 100 mg/kg), or a combination of IP injected olaparib with HO-3867 in the chow. Control mice were fed normal chow and IP injected with 200 µl of vehicle (4 %DMSO, 30 % PEG300, 66 % PBS). Treatment started three weeks post OVCAR3-RLuc injection. Tumor burden was monitored weekly by bioluminescence imaging and quantification of total flux in equal sized abdominal ROIs. To adjust for between-mouse variability in pre-treatment tumor burden, change in tumor burden was calculated by normalizing the tumor burden of each mouse at each treatment timepoint to the tumor burden of the mouse pre-treatment. Mice were monitored daily and weighed once a week per protocol to ensure no significant weight loss (>20 %) occurred and all mice were sacrificed at the end of the experiment. A full toxicity screen of HO-3867 in nude mice was prevoiusly performed by another group, highlighting the safety of this agent in a mouse model [22]. Frozen tumor tissue was processed at the University of New Mexico’s Human Tissue Repository (HTR) for sectioning and H&E staining. Slide images were processed in Aperio ImageScope software.

### RNA sequencing and analyses

Cells were seeded on 96-well culture plates at 5000 cells per well and grown for 24 h. After 24 h, cells were treated with either DMSO control, HO-3867, or APR-246. After 24 h of treatment, RNA was extracted using the Qiagen RNeasy Mini Kit (#74,104). The RNA concentration was obtained by the Qubit Fluorometer. Four µL of total RNA (200 ng/µL) was processed in the Analytical and Translational Genomics Core at the University of New Mexico. The RNA was ribo depleted with the Ribo Gone kit, and cDNA was constructed with the SMARTer Universal Low Input Kit both from Takara Bio. The libraries were then completed using the Ion Plus Fragmentation Kit (Thermo Fisher). Sequencing was conducted on Ion 550 chips in the S5XL Sequencer. The sequences were aligned to the human genome (Refseq, USCS hg19) using the RNA-Seq Alignment app version 2.0.2 using BaseSpace (Illumina). Three biological replicates were used for differential gene expression analyses, comparing each drug to DMSO control (RNA-Seq Differential Expression app version 1.0.1). Genes expressed at greater than 1.0 fragments per kilobase per million reads and with greater than 2-fold changes in gene expression were compared using Ingenuity Pathway Analysis (Qiagen). Significantly altered pathways were identified using differentially expressed genes and the WEB-based GEne SeT AnaLysis Toolkit (WebGestalt software). Illustrations of the genes impacted by drug treatment in Figs. 4A, 5A, and 6A were generated using Biorender software.

### Recombinant p53 protein expression and purification

The plasmid for WT p53 core domain (amino acid residues 94–312) in pET15b vector was obtained from Addgene (https://www.addgene.org). The protein was expressed in C41(DE3) *Escherichia coli* cells [28] in LB media. Cells were grown in a shaking incubator at 37 °C until O.D_._ 600 nm reached 0.4. Then, the incubator temperature was changed to 16 °C and protein expression was induced with 0.2 mM IPTG. The protein was purified using Talon Superflow resin (Takarabio) with a gravity-flow column. The expression of WT p53 was verified with Western Blotting using Anti-His HRP Conjugate Kits (Qiagen) visualized with SuperSignal West Pico (ThermoFisher) and ChemiDoc (Bio-Rad). The protein purity (> 80 %) was assessed by SDS-PAGE gels stained with Coomassie blue, using ImageJ (https://imagej.net/ij/).

### Ligand binding assay using florescence quenching and isothermal titration calorimetry (ITC)

The binding of HO-3867 to the purified WT p53 was confirmed by fluorescence quenching with the RF-6000 Spectrofluorophotometer (Shimadzu) and Isothermal Titration Calorimetry (ITC) with the Nano ITC (TA Instruments). For florescence quenching, 0.4 μM of purified WT p53 in a 2 mL quartz cuvette was titrated with 2 mM HO-3867 (solubilized in DMSO). The fluorescence emission spectrum (290 – 340 nm) was measured with excitation wavelength at 288 nm. The same experiment done with the DMSO control and 0.4 μM of WT p53 produced no florescence changes compared to the experiment with the protein alone. For ITC, 300 μL of 0.1 mM WT p53 (in PBS buffer with 1 % DMSO) in the reaction cell was titrated with 1.5 mM HO-3867 (solubilized with 1 % DMSO), in 21 titration steps, each of 2 µl.

### Statistical analyses

IC50 values from the cell lines were determined using GraphPad Prism software, version 9.3.1, by applying a nonlinear regression analysis (*[Inhibitor] vs. normalized response* curve) on normalized fluorescent values. Summaries of IC50 values are presented as mean values ± standard error of the mean (SEM), based from triplicate wells. Student’s unpaired *t*-test was used to determine statistical significance between treatment groups in the combination drug studies. The combinatorial index [29] was used to assess drug synergy, where values <1 suggest synergistic interactions. For the animal study, SAS software was used fit a mixed effects linear model to log-transformed fluorescence values from the region of interest in each mouse over the weeks of the study. Random intercepts were included to account for animal-specific differences at baseline, and within-animal correlations were modeled with an exponential decay autocorrelation term. Total tumor volume growth rates were estimated within groups, and among-group comparisons were made with F tests. Statistical significance was set at *p* < 0.05.

## Results

### Endometrial and ovarian cancer cell models

**Fig. 1:**
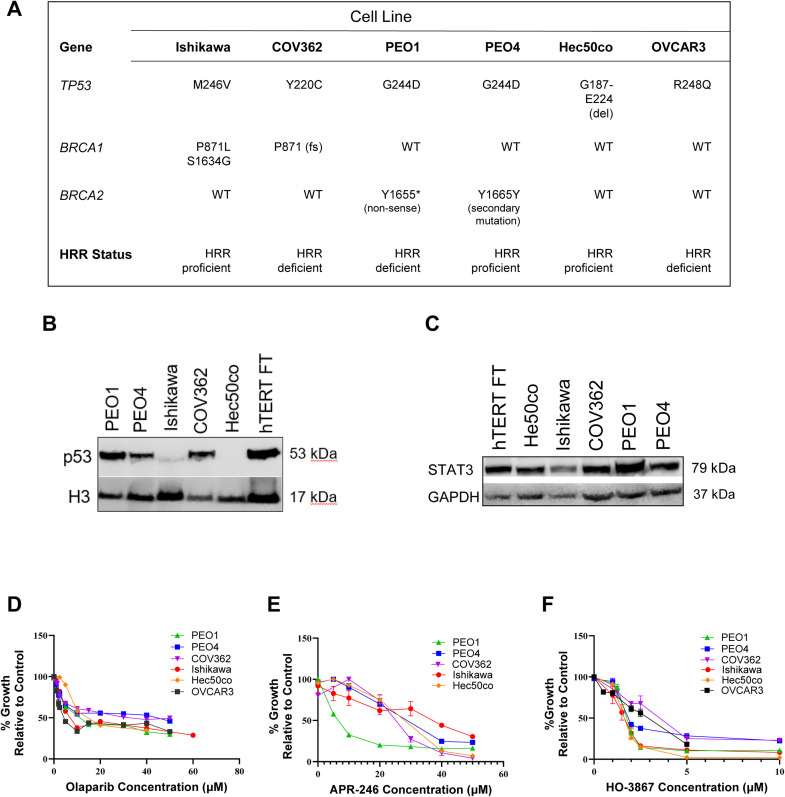
Characterization of the cell lines used in this study. A. The relevant genetic mutations and homologous recombination repair (HRR) status for each cell line. B. Western blot analysis of baseline p53 expression in each cell model. hTERT FT 194 was included as a positive control for p53 expression. Histone 3 (H3) was used as a loading control. C. Western blot of baseline STAT3 expression in each cell line. GAPDH was used as a loading control. D-F. Cell sensitivities to olaparib, APR-246, and HO-3867 after 72-h treatment. Error bars depict standard error of the mean for three replicate wells.

The gynecologic cancer models used in this study express various p53 mutants and differ by HRR status, which makes them useful for assessing the activity of p53 reactivators and PARP inhibitors [[30], [31], [32]] (Fig. 1A). Ishikawa cells are a moderately-differentiated endometrial adenocarcinoma cancer cell line harboring a heterozygous missense mutation in *TP53* (M246V), and though the effect of this mutation on p53 function has not been fully characterized, it is thought to retain at least partial WT function given its normal, low p53 expression levels [33,34]. Hec50co is a poorly differentiated serous endometrial cancer cell line with a deletion mutation in *TP53* exon 6, resulting in a p53 null phenotype [35]. COV362 cells are an endometrioid subtype of high-grade serous ovarian adenocarcinoma with the missense mutation Y220C in p53 [36]. PEO1 and PEO4 cells, which both have the missense G224D p53 mutation, were derived from a high grade serous ovarian adenocarcinoma from the same patient at different timepoints in the course of PARP inhibitor treatment. PEO1 was established when the tumor initially responded to PARP inhibitor treatment, and PEO4 cells are derived from the same patient when her tumor became PARPi resistant, which occurred when the original mutation in *BRCA2* (Y1655* nonsense mutation) reverted to BRCA WT function [37]. Given BRCA1/2 play a major role in HRR, these cells provide useful model to assess the effect of HRR function on p53 reactivation. OVCAR3 cells express the common R248Q oncogenic missense mutated form of p53 and are reported to be HRR deficient, including harboring a mutation in *BRCA2* [38]. Western blot analyses confirmed the unregulated overexpression of missense p53 in PEO1, PEO4, and COV362 cells, while p53 expression in Ishikawa was maintained at a low level consistent with WT expression, and no p53 was expressed in Hec50co cells (Fig. 1B). Since HO-3867 is known to bind directly to STAT3 as a target in addition to p53 [22], STAT3 protein expression was assessed and found to be highly expressed in most cell lines including p53 null Hec50co cells (Fig. 1C).

### Gynecologic cancer cells are highly sensitive to the curcumin analogue HO-3867 as compared to olaparib and APR-246

To assess cell sensitivities to p53 reactivator drugs, we treated five cell lines with HO-3867 or APR-246 for 72 h and measured proliferation using the CyQUANT assay. We compared these agents to the PARP inhibitor olaparib using the same experimental method. Olaparib IC50s ranged from 11.7 µM - 36.3 µM (Fig. 1D), APR-246 IC50s ranged from 5.9 µM – 48 µM (Fig. 1E), while HO-3867 IC50s were about ten-fold lower, ranging from 1.5 µM to 3.3 µM (Fig. 1F). Hec50co, which is p53 null, was the most resistant cell line to APR-246, although Hec50co was sensitive to HO-3867, suggesting that APR-246 may act predominantly through p53 reactivation, while HO-3867 has both p53-dependent and p53-independent activity. Nevertheless, as previously reported [9], we have confirmed that HO-3867 binds directly to p53. As HO-3867 is a newer compound than APR-246, and has not yet been used in clinical trials, it was important to further confirm its activity as a ligand to WT p53. Confirmation of HO-3867 binding to WT p53 was first performed using fluorescence quenching. This method assesses ligand binding of the drug to the target protein using protein fluorescent residues, such as tryptophan (Trp), tyrosine (Tyr), or phenylalanine (Phe); the change in the fluorescent residue’s environment induced by ligand binding changes the fluorescence signal [[39], [40], [41], [42]]. The core domain of WT p53 has one Trp, eight Tyr and nine Phe residues. The emission fluorescence for Trp is at ∼350 nm, and for Tyr and Phe is at ∼308 nm. The protein emission signal at 308 nm is determined after sample excitation at 288 nm. The addition of the ligand decreases the protein emission signal in a concentration-dependent manner (**Supplemental Fig. S1A**), confirming ligand binding to the protein. Further assessment of binding to p53 was performed using Isothermal Titration Calorimetry (ITC), which is a biophysical, label-free analytical technique to determine the binding of molecules in solution [43,44]. ITC shows that HO-3867 binds to WT p53 sequentially, the stoichiometry of binding between HO-3867 and WT p53 is 1 to 2, indicating that HO-3867 binds to a WT p53 dimer with a dissociation constant of 2.48 μM (**Supplemental Fig. S1B**). Though these analyses confirmed binding of HO-3867 to WT p53, our cell proliferation studies did suggest its mechanism of action is beyond direct targeting of p53.

### HO-3867 is synergistic with the PARP inhibitor olaparib

**Fig. 2:**
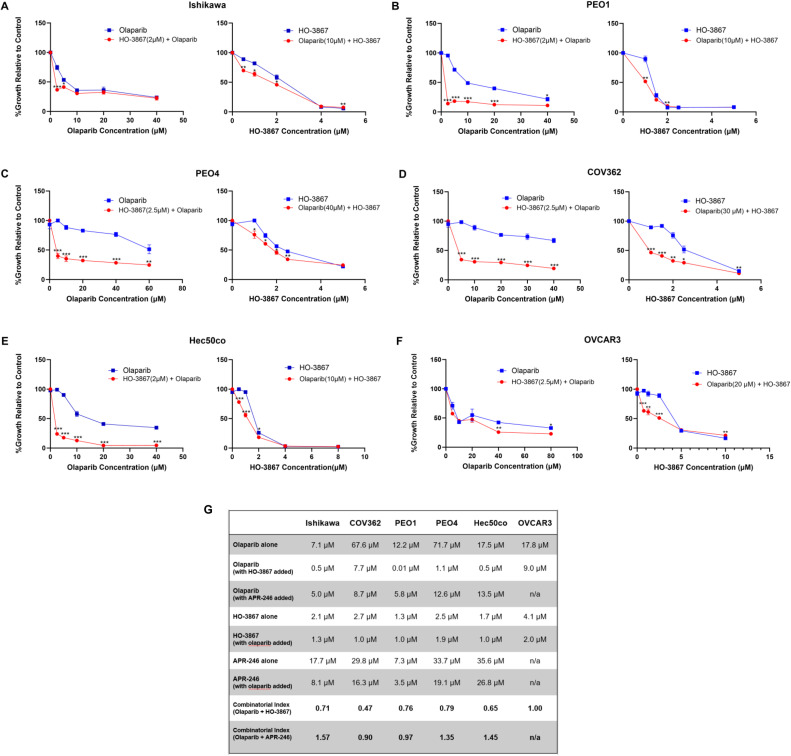
Olaparib in combination with HO-3867 is synergistic in five out of six cell lines. A.-F. HO-3867 was added at its IC50 value in combination with increasing concentrations of olaparib and compared to treatment with olaparib alone (left panels). Panels on the right depict the addition of olaparib at its cell specific IC50 value to increasing concentrations of HO-3867 and compared to HO-3867 alone. Cell proliferation after treatment for 72 h was measured using the CyQUANT assay. Error bars depict standard error of the mean for three replicate wells; *, P<0.05; **, P<0.01; ***, P<0.001. G. IC50 values, obtained from cell proliferation experiments, for olaparib and p53 reactivators alone and in combination. Combinatorial index values are also denoted, where values <1 indicate synergy, values >1 indicate antagonism and values equal to 1 indicate additive effects.

**Fig. 3:**
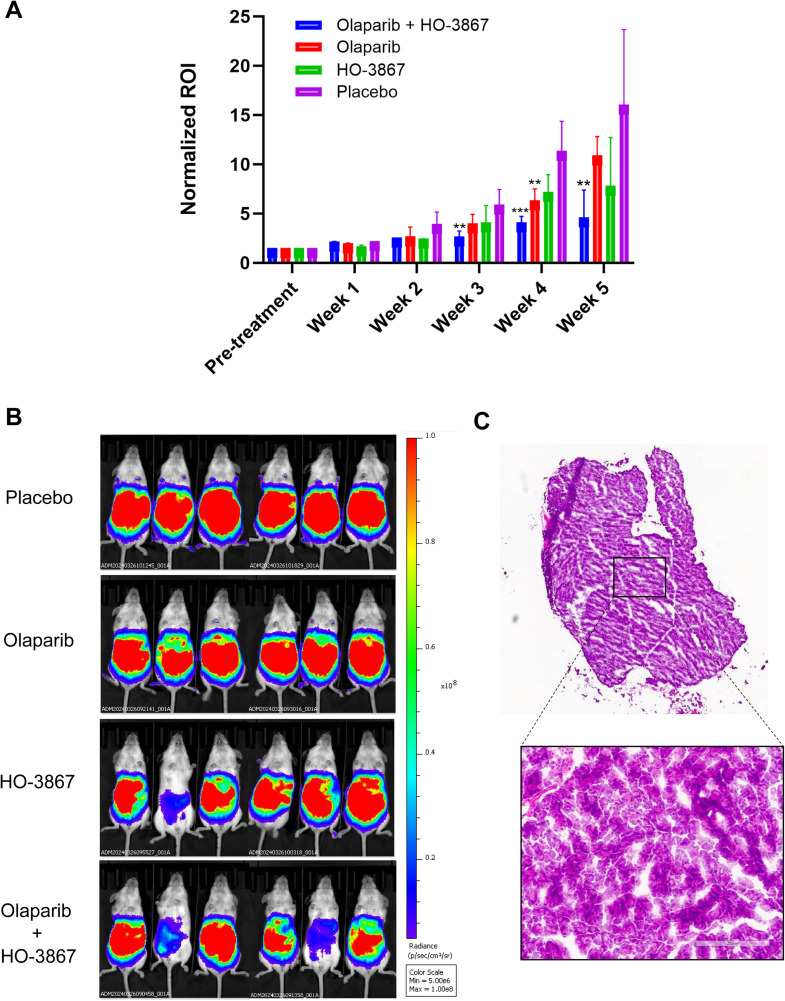
HO-3867 in combination with olaparib significantly reduces OVCAR3 tumor growth in a mouse model. OVCAR3 cells were transduced with luciferase and injected IP into NSG mice. Once tumors were established in the pre-treatment interval, animals were divided into groups and given either HO-3867 (P.O), olaparib (IP injection), both, or a placebo, for five weeks. A. Animals were imaged on the IVIS Spectrum *in vivo* imaging system, and the total tumor burden, based on bioluminescence, was measured. Total flux/mouse was normalized to pre-treatment total flux to quantify change in tumor burden/week. Treatment groups with significantly reduced tumor burden compared to the placebo group are indicated; *, *P* < 0.05; **, *P* < 0.01; ***, *P* < 0.001. Error bars represent standard deviation. B. Bioluminescence images of total tumor burden acquired on IVIS Spectrum after five weeks of treatment. C. Hematoxylin & eosin (H&E) stain of OVCAR3 tumor extracted from a mouse in the placebo group.

To investigate whether p53 reactivators enhance olaparib treatment, we tested the co-treatment of HO-3867 or APR-246 with olaparib and obtained IC50s from cell growth studies. We found that the addition of p53 reactivators lowered IC50 concentrations of olaparib in most of the cell lines studied. Strikingly, we found synergistic effects between HO-3867 and olaparib in all cell lines except OVCAR3, where the combination showed additive effects (Figs. 2A-F). The combinatorial index (CI) values for HO-3867 and olaparib were: Ishikawa, 0.71; COV362, 0.47; PEO1, 0.76; PEO4, 0.79; Hec50co, 0.65; OVCAR3, 0.99 (Fig. 2G). There did not appear to be a synergistic or additive effect of adding APR-246 in most cells except PEO1 and COV362, which were modestly more sensitive to olaparib when APR-246 was added (**Supplemental Fig. S3**). Interestingly, antagonism was detected between olaparib and APR-246 in the three other cell lines, Ishikawa, PEO4, and Hec50co. and CI values for APR-246 and olaparib were: Ishikawa, 1.57; COV362, 0.90; PEO1, 0.97; PEO4, 0.1.35; Hec50co, 1.45 (Fig. 2G). In addition, the combination of HO-3867 and olaparib was more effective in limiting tumor growth in the OVCAR3 xenograft model compared to either agent given individually (Figs. 3A-C **and Supplemental Fig. S2**).

### Tumor suppressive p53 transcriptional effects are reinstated after HO-3867 and APR-246 treatment

**Fig. 4:**
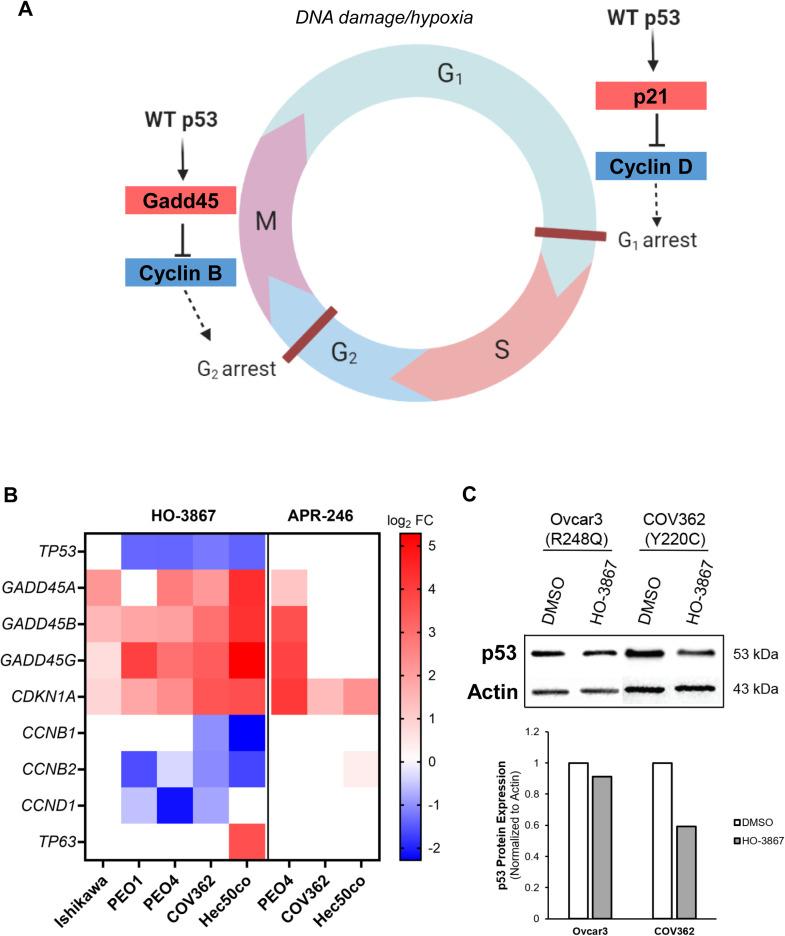
Cell cycle control typical of WT p53 function is reinstated with HO-3867 treatment. A. Diagram of WT p53 control of cell cycle checkpoints. B. Heat map of cell cycle genes that were significantly upregulated (red) or downregulated (blue) in response to treatment with HO-3867 or APR-246 for 24h. Scale bar represents log_2_ fold change values. Ishikawa and PEO1 had no significant gene changes after APR-246 treatment. C. Western blot and quantitation of p53 expression after treatment for 24h with HO-3867 in COV362 and OVCAR3 cells.

Next, we explored mechanisms of action of the two p53 reactivator agents, HO-3867 and APR-246, using bulk RNA sequencing. We treated cells with HO-3867 or APR-246 at their IC50 concentration and extracted RNA after 24 h to identify transcriptional changes compared to control cells. After HO-3867 treatment, there was a significant upregulation of genes encoding p21 (*CDKN1A*) and Gadd45 (*PPP1R15A*) and downregulation of genes encoding Cyclin B (*CCNB1/2*) and Cyclin D (*CCND1*) (Fig. 4A), indicating a reinstatement of cell cycle regulation normally driven by WT p53 [45]. Additionally, expression of *TP53* was significantly downregulated in all cell lines with oncogenic p53 expression, suggesting a restoration of the protein’s negative feedback loop wherein WT p53 downregulates its own expression [46]. After APR-246 treatment, *TP53* transcript levels were unchanged, though as with HO-3867, some cell lines showed an induction of *CDKN1A* and *PPP1R15A* genes, which are established downstream WT p53 target transcripts (Fig. 4B). Interestingly, PEO1 and Ishikawa had no significant gene expression changes in response to APR-246. We verified the downregulation of p53 protein after HO-3867 treatment via western blot, shown in Fig. 4C.

**Fig. 5:**
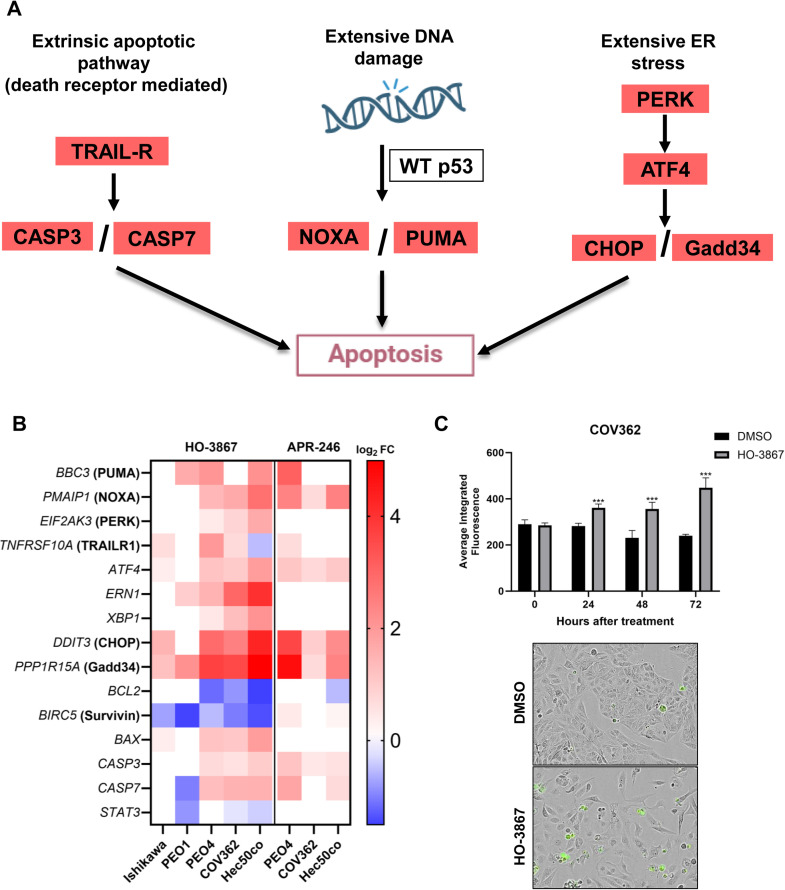
Major genes in apoptotic pathways significantly affected by p53 reactivators. A. A diagram representing major apoptotic pathways with genes found to be upregulated by p53 reactivators highlighted in red. B. Heatmap of genes involved in the ER stress pathway that were significantly upregulated (red) or downregulated (blue) in response to 24-h treatment with HO-3867 or APR-246. Scale bar represents log_2_ fold change values. Ishikawa and PEO1 had no significant gene changes in response to APR-246. C. COV362 cells were treated with DMSO control or HO-3867 (4 μM) for up to 72h and caspase activity was measured using the CellEvent Caspase-3/7 Assay. Total fluorescence in each well was measured using the Incucyte software’s calculated average integrated fluorescence. Error bars represent standard error of the mean for four replicate wells. Representative images are from the Incucyte at 72h after treatment.

In addition to cell cycle checkpoint reinstatement, RNA seq results reveal that apoptosis is a major mechanism of cell killing by these agents. This is most evident in cells treated with HO-3867, where p53 pro-apoptotic target genes *PMAIP1* (NOXA) and *BBC3* (PUMA) were significantly upregulated in most cell lines (Figs. 5A, 5B). NOXA and PUMA transcripts were also upregulated after APR-246 treatment, though to a lesser extent than after HO-3867 treatment (Fig. 5B).

### p53-independent pathways are induced after HO-3867 and APR-246 treatment

Beyond p53 binding, HO-3867 has been shown to bind directly to STAT3 and inhibit its phosphorylation and DNA binding [22]. STAT proteins are commonly upregulated in cancer cells as they promote cell growth and inhibit apoptosis [47]. Thus, the inhibition of STAT3 is an important anti-tumor approach and likely one of the major mechanisms through which HO-3867 is inhibiting cell growth in these experiments. In p53-null Hec50co cells, the *STAT3* transcript was reduced after HO-3867 treatment, and direct transcriptional targets of STAT3, including pro-survival genes *BCL2* and *BIRC5* (Survivin) [48], were also downregulated in response to HO-3867 (Fig. 5A). These results suggest that in p53 null Hec50co cells, HO-3867 may be acting through inhibiting STAT3. *BCL2* was also downregulated in response to APR-246 in Hec50co cells, but no other cell models exhibited the downregulation of *BCL2* or *BIRC5* after APR-246 treatment, indicating that STAT3 signaling was less impacted by APR-246 compared to HO-3867.

In addition, other apoptotic pathways that function independently of p53 activity [49], including receptor mediated (extrinsic) apoptosis and ER-stress mediated apoptosis, were upregulated in response to HO-3867 and APR-246 (Fig. 5A). The death receptor TRAIL-R (*TNFRSF10A*) and Caspases 3 and 7, which are major components of the extrinsic apoptotic pathway, were induced in several cell lines in response to both HO-3867 and APR-246 (Fig. 5B). The activation of caspase 3/7-mediated apoptosis in response to HO-3867 was confirmed in a live-cell assay in COV362 cells (Fig. 5C). Further, several genes critical to the ER-stress mediated pathway, including PERK *(EIF2AK3), EIF2A, ATF4,* Gadd34 (*PPP1R15A*), and CHOP (*DDIT3*)*,* which directly activates apoptosis, were all upregulated. For HO-3867, most of these genes were induced in all cell lines except PEO1, and none of these ER pathway genes were altered in response to APR-246 in either PEO1 or Ishikawa cells. Interestingly, Hec50co cells were the only cells to upregulate the p53 family member gene *TP63* after HO-3867 treatment, suggesting p63 may be acting as a backup tumor suppressor in the absence of p53 (Fig. 5B). The complete list of pathways most highly induced and downregulated in response to HO-3867 and APR-246 are depicted in **Supplemental Figs. S4 and S5.**

### Members of the HSP70 family are highly induced after HO-3867 treatment

Strikingly, *HSPA6* was one of the most highly upregulated genes in all cell lines after both HO-3867 and APR-246 exposure, with log_2_ fold changes as high as 12.1, and other HSP70 family members, including *HSPA7, HSPA1A, HSPA1B*, were also very highly induced (**Supplemental Fig. S6**). The HSP70 protein family members have several functions critical to proteostasis including acting as chaperones in protein folding and multi-protein complex assembly [50]. The upregulation of HSPs is a general stress response to promote cell survival [50], though the extreme upregulation of HSP70 family members in response to p53 reactivator treatment may be acting as chaperones to refold mutant p53 [51]. In support of this observation, it is reported that HSPA6 is crucial to the proper folding of WT p53 and has been shown to enhance p53 binding to the p21 promoter after heat-shock induced stress [52,53].

### DNA repair pathways are inhibited by HO-3867

**Fig. 6:**
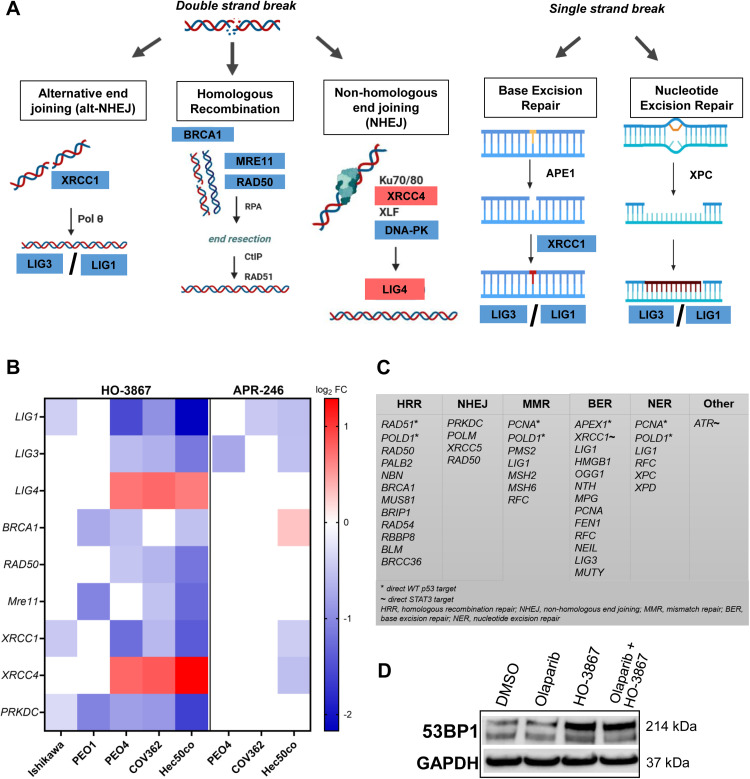
HO-3867 represses multiple DNA repair genes and causes DNA damage. A. A diagram of major DNA repair pathways and genes affected by HO-3867. B. Heat map of DNA repair gene expression changes after cells were treated with HO-3867 or APR-246 treatment for 24h. Scale bar represents log_2_ fold change values. Ishikawa and PEO1 cells had no significant gene changes after treatment with APR-246. C. A list of DNA repair transcripts downregulated in response to HO-3867 in PEO4 cells. D. Western blot showing 53BP1 expression in COV362 cells treated with DMSO control, olaparib, HO-3867, or the combination of olaparib and HO-3867, for 24h. GAPDH was used as a loading control.

Another effect of these agents was the inhibition of multiple DNA repair pathways, including HRR, alternative end joining, base excision repair and nucleotide excision repair (Fig. 6A). This effect was primarily seen in cells treated with HO-3867, though APR-246 also resulted in the downregulation of some DNA repair genes including *LIG1* and *LIG3* (Fig. 6B). WT p53 is known to repress the transcription of specific DNA repair genes, including *RAD51* [*54*], *POLD1* [*55*], *PCNA* [56], and *APEX1* [57], all found to be downregulated in PEO4 cells after HO-3867 treatment (Fig. 6C). Additionally, the activation of STAT3, which is upregulated in cancer cells, has been shown to enhance DNA repair [58]. STAT3 is a confirmed target of HO-3867, and we found the STAT3-controlled genes *XRCC1* [59] and *ATR* [58] to be downregulated in PEO4 cells, identifying another likely mechanism of DNA repair inhibition that affects both HRR deficient and HRR proficient cells. Western blotting confirmed the downregulation of DNA repair proteins LIG1, NBN, and XRCC1 in PEO4 cells treated with HO-3867 (**Supplemental Fig. S7**) and, notably, 53BP1 protein was highly induced after HO-3867 treatment in COV62 cells (Fig. 6D), highlighting the presence of DNA damage as a result of HO-3867. Interestingly, PEO4, COV362, and Hec50co cells upregulated the expression of *LIG4* and *XRCC4*, which are involved in NHEJ, indicating a possible compensatory repair mechanism being utilized in these cells.

### Immunomodulatory pathways are impacted by HO-3867

Oncogenic mutations in p53 inhibit T-cell tumor recognition through enhanced PD-L1 and PD-L2 tumor cell expression, thereby inhibiting T-cell immune recognition [[60], [61], [62]]. In these studies, HO-3867, but not APR-246, resulted in alterations to the immunomodulatory transcriptome that predict for enhanced tumor recognition by CD8+ *T* cells. MHC Class 1 genes, including *HLA-A, HLA-B*, and *HLA-C, IFNGR,* and immuno-proteosome subunit genes *PSMA* and *PSMB,* were significantly upregulated in cells (**Supplemental Fig. S6**). In contrast, these immunological pathways were not impacted by APR-246, although both agents induced the antigen processing pathway as defined by WEB-based GEne SeT AnaLysis Toolkit (WebGestalt software, **Supplemental Fig. S4 and S5**).

## Discussion

*TP53* is the most frequently mutated gene in all cancer types and its loss of normal function characterizes the most aggressive ovarian and endometrial cancers. Unfortunately, current treatment strategies have shown limited efficacy for patients with these tumor types, as they often acquire resistance to chemotherapy or PARP inhibitors. Our findings in this study shed light on a new promising therapeutic strategy that aims to target misfolded p53 and induce cell death. Of the two p53 reactivator drugs tested, HO-3867 and APR-246, we found the novel curcumin analogue HO-3867 to be superior to APR-246 in inhibiting ovarian and endometrial cancer cell growth. HO-3867 showed more clinically relevant IC50 values as compared to APR-246, and synergistic effects were observed when combined with the PARP inhibitor olaparib *in vitro* and *in vivo* in a xenograft model of advanced ovarian cancer with mutant p53.

Improving PARP inhibitor therapy effectiveness is an urgent need for patients [63]. While our studies showed that co-treatment of olaparib with HO-3867 is synergistic, the effect of APR-246 in combination with olaparib is more modest and selective; cells with impaired HRR showed improved sensitivity to olaparib, while cells with functional HRR showed no sensitivity difference when APR-246 was added. Cells that are HRR deficient have higher genomic instability after PARP inhibition, as they rely on low-fidelity DNA repair pathways [64]. Transcriptome analyses revealed the drastic downregulation of multiple DNA repair pathways after HO-3867 treatment, resulting in extensive DNA damage as indicated by induction of protein p53-binding protein 1 (53BP1) after HO-3867 exposure. 53BP1 is recruited to double strand breaks and plays a lead role in regulating downstream repair pathways, specifically by promoting non-homologous end joining (NHEJ) via blocking strand resection and preventing homologous recombination [65]. This provides a likely mechanism explaining the synergy of HO-3867 and olaparib co-treatment even in HRR proficient cells. These findings support the potential of HO-3867 and, to a lesser extent, APR-246, to be effective in improving PARP inhibitor therapy, with HO-3867 being applicable more broadly to include HRR proficient and p53 null tumors.

Both drugs reinstated classic WT p53 transcriptional effects, including the induction of p21, NOXA and PUMA. HO-3867 also induced the downregulation of *TP53* itself, which is a critical therapeutic effect in cells addicted to oncogenic mutant p53. While some studies have identified mechanisms of APR-246 action beyond direct p53 binding in cancers [66], our data demonstrate that one of its primary modes of action is targeting the p53 pathway [18], as APR-246 was largely ineffective in p53 null cells compared to HO-3867. While HO-3867 is also classified as a “p53 reactivator”, confirmed in the present study to directly bind p53 and induce expression of p53 target genes, HO-3867 clearly acts beyond p53, including the previously discovered direct binding to STAT3 [22]. Given the high expression of STAT3 in our cell models and downregulation of the STAT3-controlled genes *BCL-2, Survivin, XRCC1,* and *ATR* after HO-3867 exposure, we propose that inhibition of STAT3 mediated pro-growth effects is likely a mechanism of HO-3867 action in our study. Further, the broad repression of DNA repair pathways including the downregulation of homologous recombination DNA repair genes directly controlled by WT p53 and STAT3, likely contributes to enhanced olaparib sensitivity when HO-3867 is added, even in HRR proficient cells.

Importantly, STAT3 inhibits WT *TP53* transcription by binding to its DNA promoter and, in a reciprocal manner, WT p53 limits the activity of STAT3 through a phosphorylation event on a tyrosine that is required for STAT3 transcriptional activity [67]. Accordingly, STAT3 activity is highly induced in p53 null cell models such as Hec50co cells, as well as in many p53 missense mutated cancer cells. As STAT3 activity is typically robust in p53 mutant cancers, whether they be p53 null or p53 missense cases, the dual targeting of both p53 and STAT3 by HO-386 provides a therapeutic advantage over other p53 reactivator agents and expands the usefulness of this agent even in p53 null cases.

In addition to the activation of p53-dependent apoptosis, we identified p53-independent apoptotic pathways that were upregulated after treatment with HO-3867 and APR-246. Genes critical to receptor-mediated (extrinsic) apoptosis, including *TRAIL-R*, as well as downstream caspase genes, *CASP3* and *CASP7,* were induced after exposure to both agents. The ER-stress mediated apoptotic pathway was also upregulated after APR-246 and HO-3867 exposure. Through this pathway, upon ER stress, PKR-like ER kinase (PERK, a product of the *EIF2AK3* gene) is activated and phosphorylates eukaryotic translation initiation factor 2A (eIF2α) to attenuate translation. Phosphorylated eIF2α also promotes the translation of activating transcription factor 4 (ATF4) which activates C/EBP homologous protein (CHOP, a product of the *DDIT3* gene) and Gadd34 (*PPP1R15A*) transcription. If ER stress fails to resolve, CHOP will activate apoptosis [68]. Interestingly, this pathway is shown to require p63 and PUMA, not p53 [69], and both *TP63* and *BBC3* (PUMA) were significantly induced in Hec50co cells (p53 null) after HO-3867 treatment. Importantly, because *TP63* was not affected in any other cell line with mutant p53, this suggests that enhanced transcription of p63 by HO-3867, despite the lack of p53, may be a therapeutic mechanism of action of this agent in p53 null cells [70]. As a future avenue for research, there is a class of “p63 activation drugs” under study in other cancers that enhance p63-mediated tumor suppressive activity [71]. Such agents could be important therapeutic drugs in tumors with mutant p53 [72].

Strikingly, we found the HSP70 family genes to be highly induced by both agents upon treatment. Specifically, the HSP70 family member *HSPA6* was one of the most highly induced genes in all cell lines after HO-3867 and APR-246. This effect was observed even in cells that are p53 null. Heat shock proteins serve as chaperones for mis-folded proteins and may be induced in this setting to re-fold mutant p53. More generally, however, HSPs are induced by severe cell stress and help the cell to cope with an accumulation of denatured proteins [73], thus, they may be playing a protective role against the drug’s cytotoxicity. Further studies are needed to determine the precise role of HSP70s. The induction of HSPs may be either a therapeutic mechanism of drug activity, or on the other hand, may signify cell resistance to treatment.

Finally, we found evidence supporting the ability of HO-3867 to enhance immune system activation. Genes encoding MHC Class I expression and the immunoproteasome were induced, predicting increased CD8+ *T* cell tumor recognition. Immunotherapy is an important new FDA approved treatment approach that has become part of the standard of care for endometrial cancers [74,75], and p53 reactivators have the potential to further enhance immunotherapy in the treatment of endometrial cancer. It is well known that ovarian cancers do not respond robustly to PD-1/PDL-1 inhibitors in comparison to endometrial cancers. One possibility is the high prevalence of mutant p53 proteins that inhibit CD8+ *T* cell tumor recognition [[60], [61], [62]]. Reactivation of p53 functionality in ovarian cancers has the potential to sensitize tumor cells to immunotherapy, and we propose that this should be a future area of intense research.

Some limitations of this study include the focus on cell line models, which do not always translate to what occurs in patient tumors in terms of drug response. Additionally, the RNA sequencing analyses were exploratory, requiring additional studies to confirm the proposed mechanisms of drug actions. Nevertheless, our findings highlight the promising role of p53 reactivators, specifically the curcumin analogue HO-3867, in expanding the repertoire of cases that are sensitive to p53 reactivation and PARP inhibitor treatment. First, HO-3867 restores tumor suppressor function to otherwise oncogenic missense mutant p53. HO-3867 is effective at low IC50 concentrations in preclinical *in vitro* and *in vivo* models and is widely synergistic with olaparib, effects that were observed not only in HRR deficient, p53 missense mutated cells, but surprisingly, also in HRR proficient and p53 null cells. Second, HO-3867, but not APR-246, is synergistic with the PARP inhibitor olaparib by down regulating multiple DNA repair pathways used by cancer cells to escape the effects of PARP inhibition. These findings highlight the potential of certain curcumin analogues such as HO-3867 to prevent resistance to PARP inhibitors, and this concept should be studied in future clinical trials.

## CRediT authorship contribution statement

**Lane E. Smith:** Writing – original draft, Methodology, Investigation, Formal analysis, Data curation. **Jamie L. Padilla:** Methodology, Investigation, Formal analysis, Data curation. **Angelina Licor:** Investigation. **Mara P. Steinkamp:** Visualization, Supervision, Resources, Project administration, Methodology, Formal analysis. **Irina V. Lagutina:** Methodology, Investigation. **Yan Guo:** Supervision, Software, Formal analysis, Data curation. **Eric J. Devor:** Methodology, Investigation. **Vernon S. Pankratz:** Validation, Software, Formal analysis. **Annahita Sallmyr:** Visualization, Supervision. **Olufunmilola M. Oyebamiji:** Methodology, Formal analysis, Data curation. **Jun-yong Choe:** Methodology, Investigation, Formal analysis. **Geneva L. Williams:** Writing – review & editing, Visualization, Methodology, Investigation. **Kimberly K. Leslie:** Writing – review & editing, Writing – original draft, Validation, Supervision, Resources, Project administration, Methodology, Investigation, Funding acquisition, Formal analysis, Data curation, Conceptualization.

## Declaration of competing interest

The authors declare that they have no known competing financial interests or personal relationships that could have appeared to influence the work reported in this paper.

## Appendix Supplementary materials

Image, image 1

Image, image 2

Image, image 3

Image, image 4

Image, image 5

Image, image 6

Image, image 7
