# Supplementary figures and images for: Novel p53 reactivators that are synergistic with olaparib for the treatment of gynecologic cancers with mutant p53
Source: Transl Oncol. 2025 Sep 6;61:102522. doi: 10.1016/j.tranon.2025.102522 (PMC12450570; doi:10.1016/j.tranon.2025.102522)

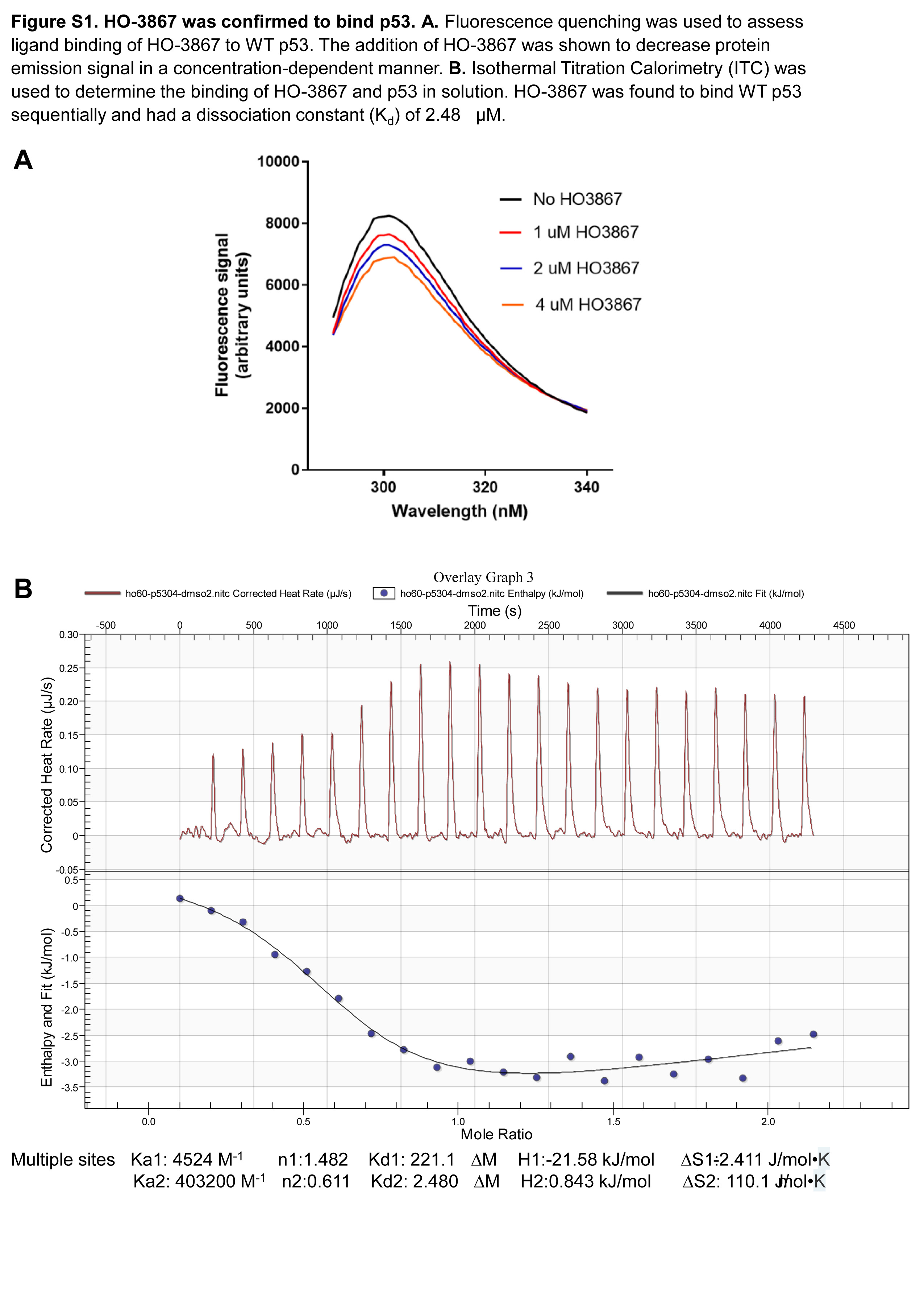

Supplement: Supplementary file 1 [file mmc1.jpg]

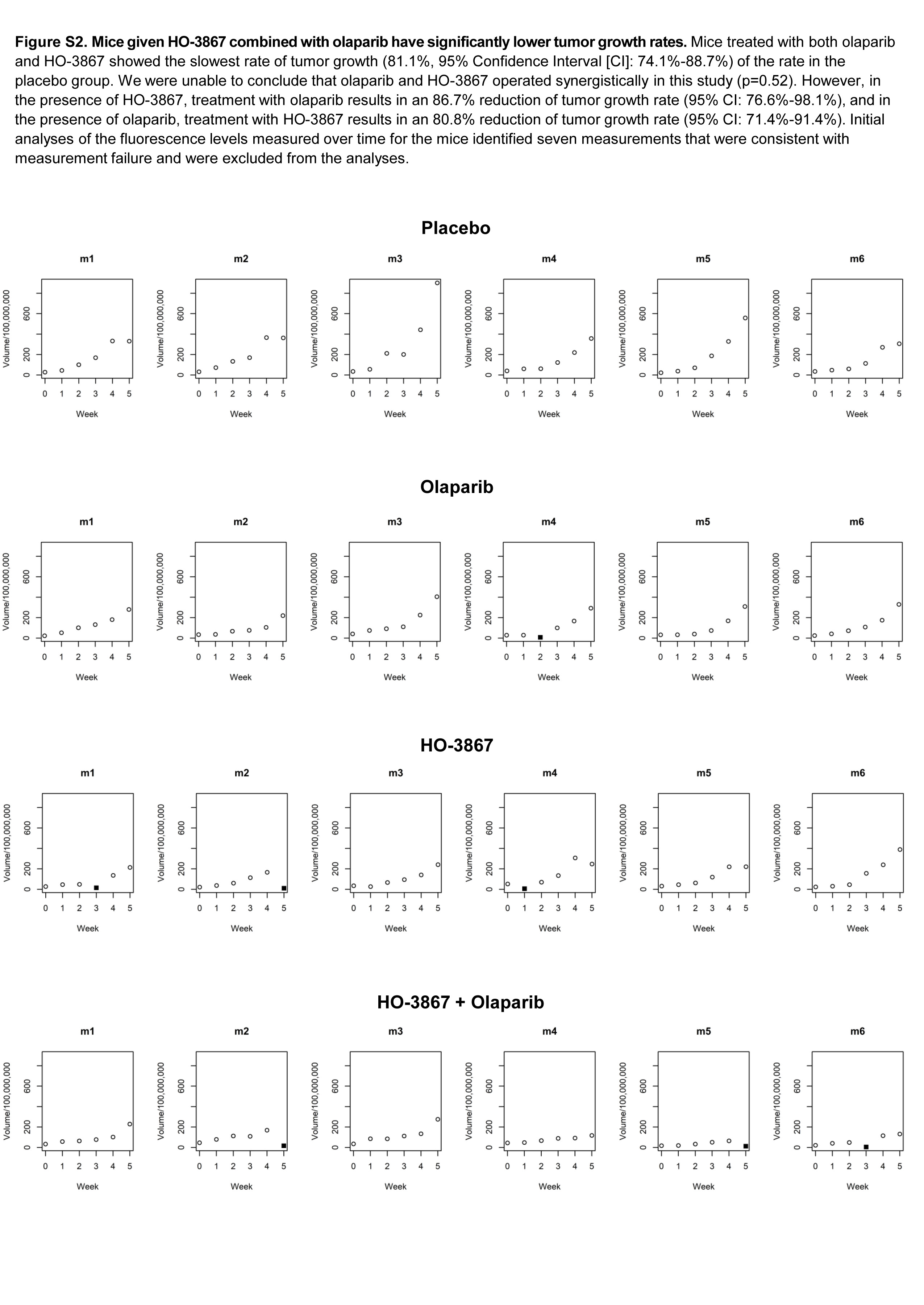

Supplement: Supplementary file 2 [file mmc2.jpg]

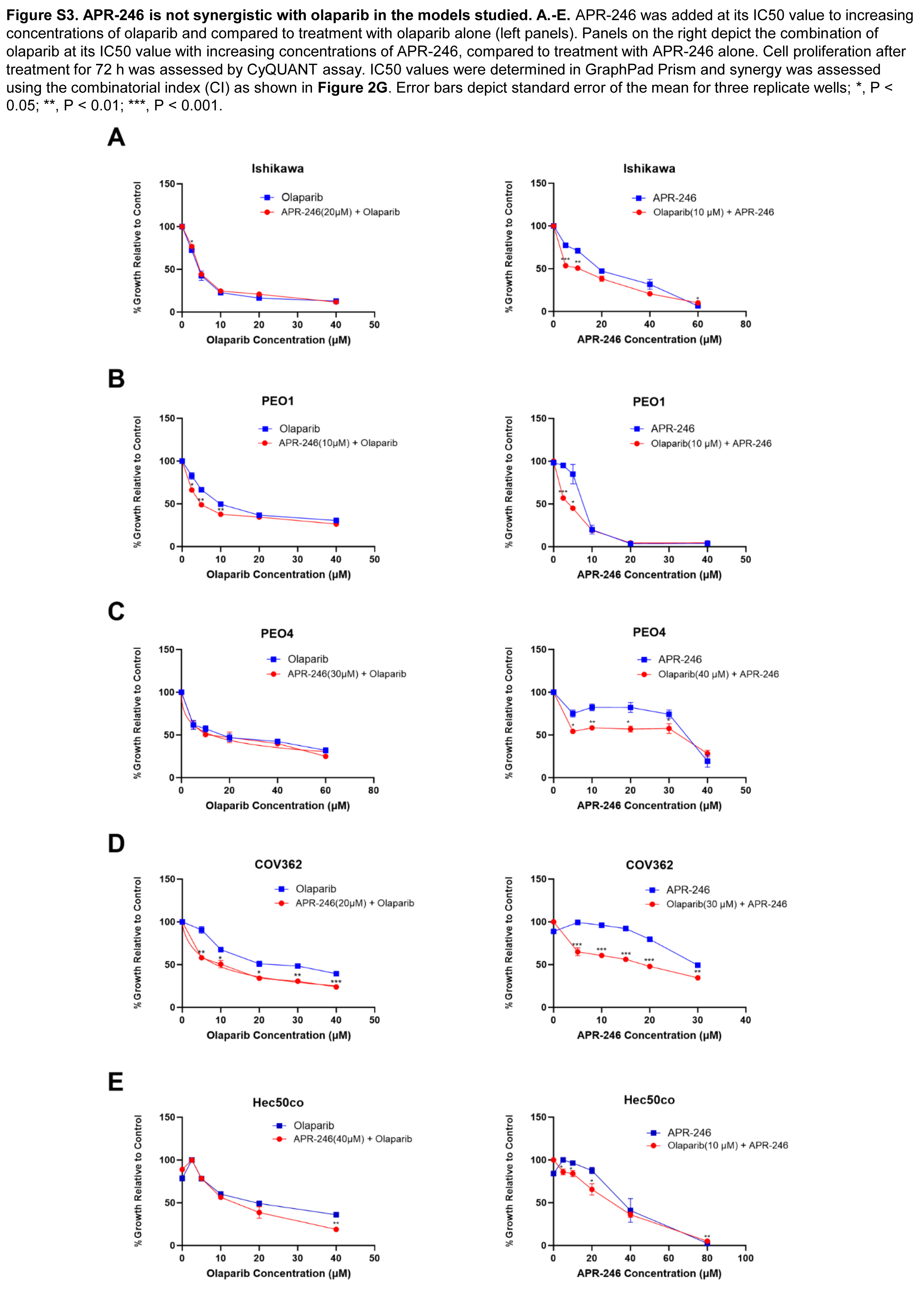

Supplement: Supplementary file 3 [file mmc3.jpg]

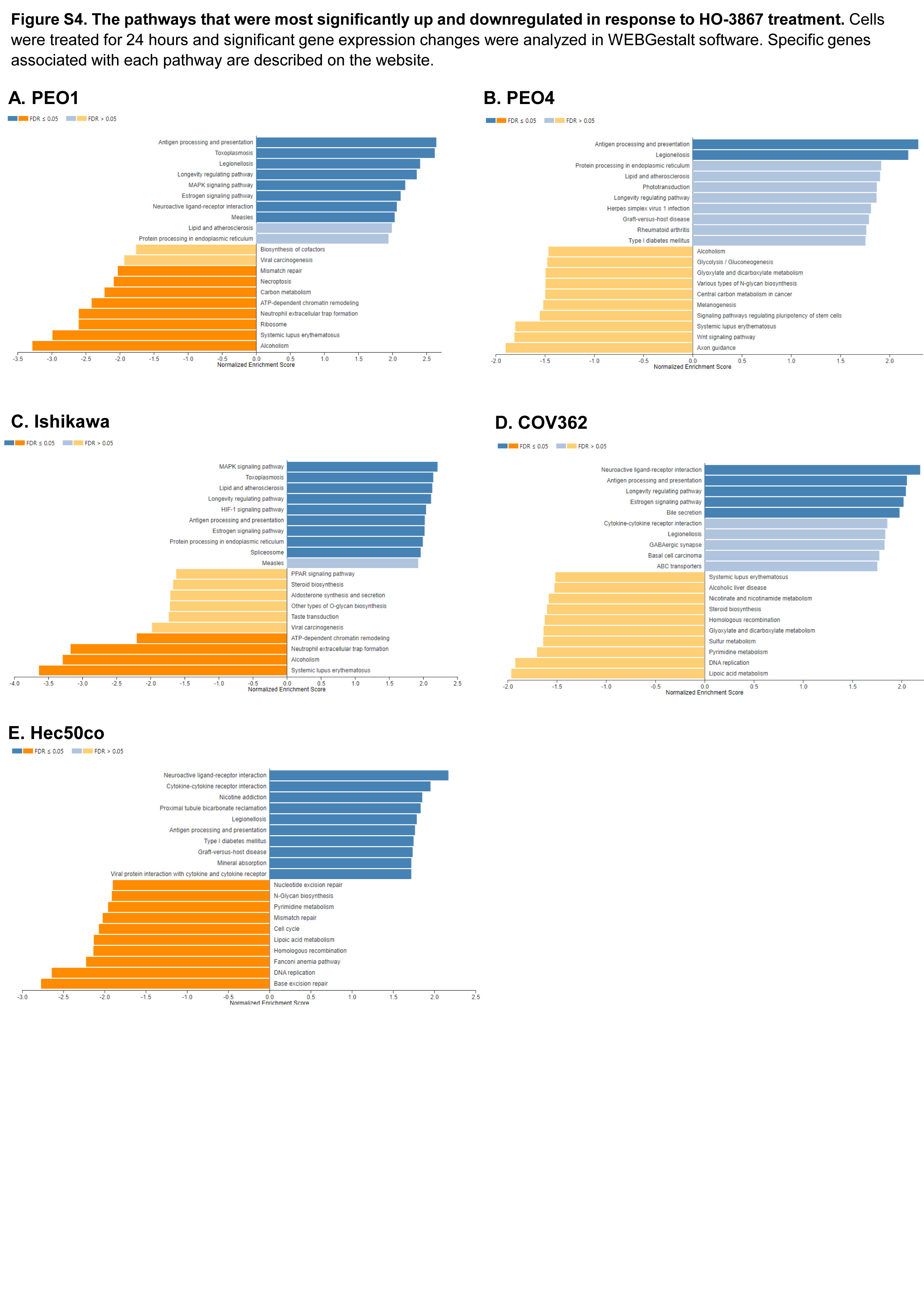

Supplement: Supplementary file 4 [file mmc4.jpg]

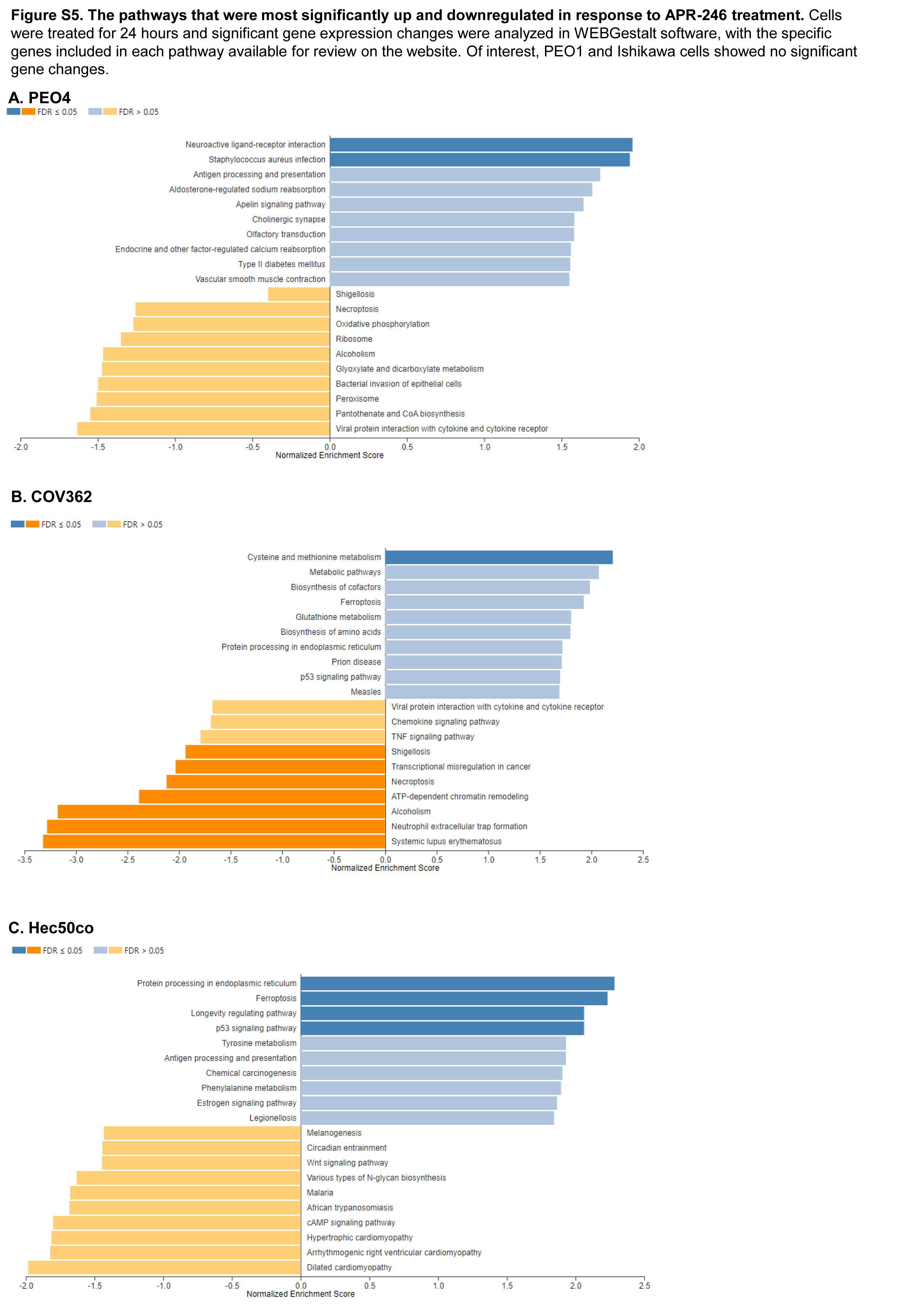

Supplement: Supplementary file 5 [file mmc5.jpg]

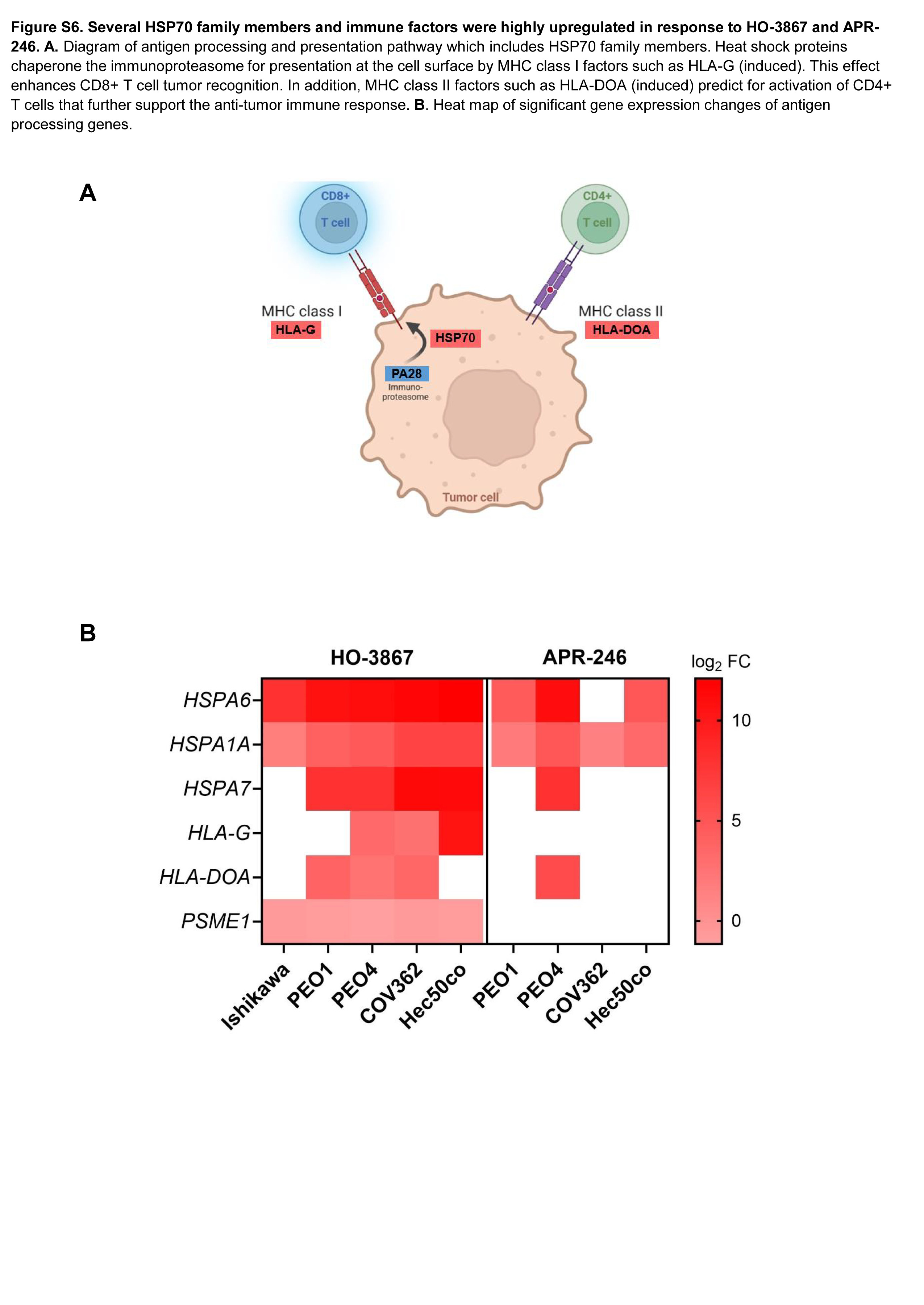

Supplement: Supplementary file 6 [file mmc6.jpg]

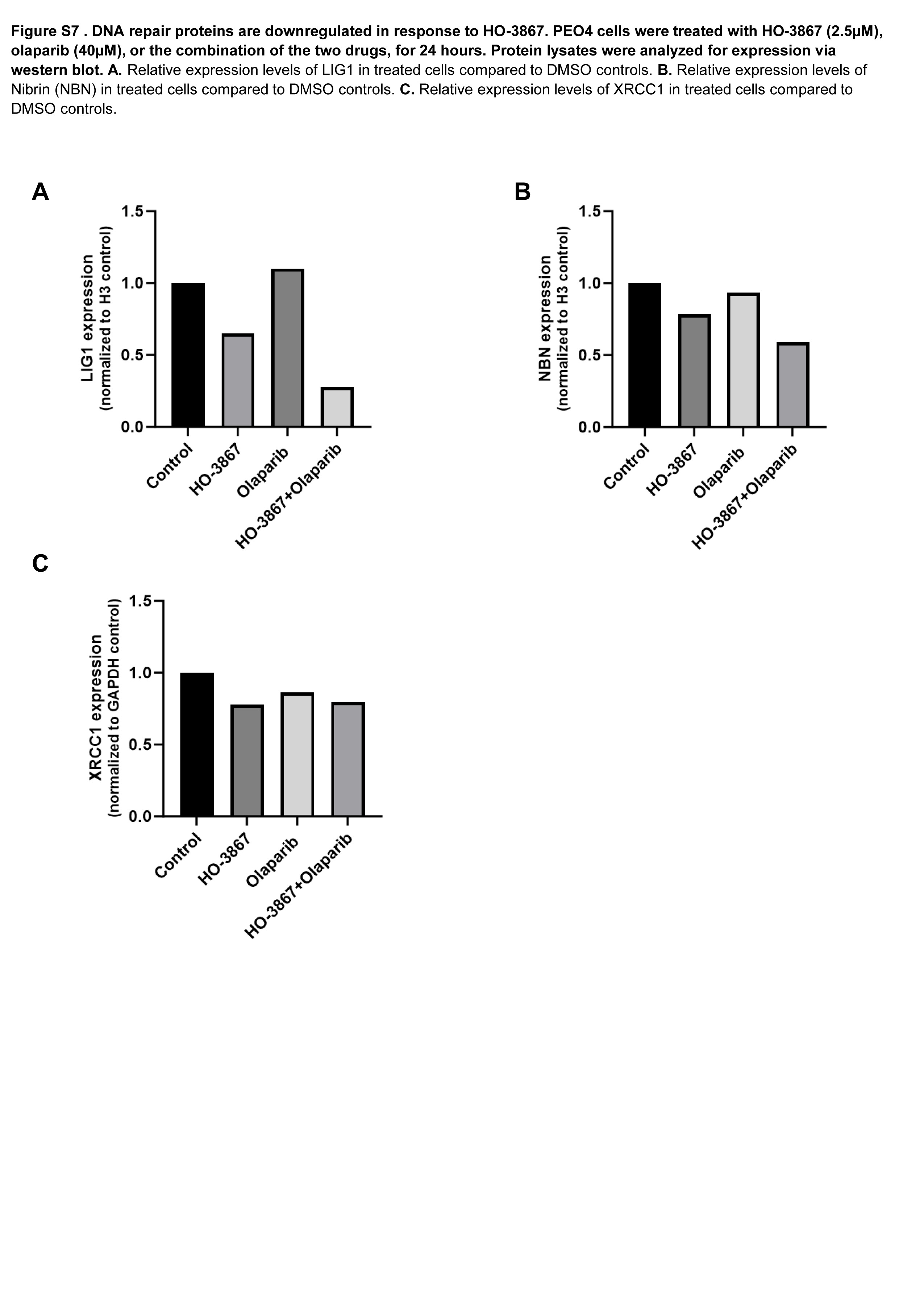

Supplement: Supplementary file 7 [file mmc7.jpg]
